# Association between forage mycotoxins and liver disease in horses

**DOI:** 10.1111/jvim.16486

**Published:** 2022-07-06

**Authors:** Andy E. Durham

**Affiliations:** ^1^ The Liphook Equine Hospital Liphook United Kingdom

**Keywords:** fumonisin B1, hay, hepatic, outbreak, toxicity

## Abstract

**Background:**

Outbreaks of liver disease in horses are common but the etiology of most remains unknown. Forage mycotoxins have been suspected to be a cause.

**Objectives:**

To examine the association between outbreaks of liver disease and the presence of mycotoxins in forage stored on the same premises.

**Animals:**

Premises were identified where ≥4 horses were contemporaneously affected by liver disease, and a control group was formed from premises where ≥4 horses had been examined and found to have no evidence of liver disease.

**Methods:**

Forage was collected from 29 case and 12 control premises. The forage was analyzed for mycotoxin content using a liquid chromatography/mass spectrometry method, targeting 54 mycotoxins. The presence and distribution of mycotoxins between case and control samples was compared.

**Results:**

Mycotoxins were found in 23/29 (79%) case samples and 10/12 (83%) control samples (*P* > .99; relative risk, 0.93; 95% confidence interval [CI], 0.64‐1.75). Median (interquartile range [IQR]) total mycotoxin concentration was similar in case and control samples (85.8 μg/kg [1.6‐268] vs. 315 μg/kg [6.3‐860]; *P* = .16). Ten mycotoxins were found exclusively in case premises comprising fumonisin B1, 15‐acetyldeoxynivalenol, deoxynivalenol, zearalenone, aflatoxins B1 and G1, methylergonovine, nivalenol, verruculogen, and wortmannin. The median (IQR) concentration of fumonisin B1 was significantly higher in case versus control samples (0 μg/kg [0‐81.7] vs. 0 μg/kg [0‐0]; *P* = .04).

**Conclusions and Clinical Importance:**

Several mycotoxins with known hepatotoxic potential were found, alone or in combination, exclusively at case premises, consistent with the hypothesis that forage‐associated mycotoxicosis may be a cause of outbreaks of liver disease in horses in the United Kingdom.

## 1 INTRODUCTION

Liver disease is commonly encountered in equine practice both as clinical and subclinical disease.^1^ Outbreaks of hepatic disease are common and once often were suspected to be caused by pyrrolizidine alkaloid (PA) toxicosis,^2^ although more recent evidence suggests that PA toxicity is far less common than generally suspected.^3^ Some other plants also have been incriminated in hepatopathy cases although incidents appear to be rare.^4^ Alternative explanations for outbreaks of hepatopathy might include infectious pathogens, and evidence recently has emerged of viral hepatitides in horses.^5^, ^6^ However, in the majority of liver disease outbreaks in horses, specific etiologic diagnoses remain elusive. Many fungi produce metabolites that possess antibacterial, antiviral, anthelmintic, antifungal, herbicidal, and insecticidal properties, which may provide a competitive advantage.^7^, ^8^ Some fungal products are also toxic to mammalian species, and >500 such mycotoxins have been identified, primarily from fungi of the genera Aspergillus, Penicillium, Alternaria, and Fusarium.^9^ Mycotoxins are a major human health concern with approximately 25% of global crop production being contaminated.^10^ Monogastric species including horses are considered more susceptible to mycotoxins than ruminants^11^ with the main groups endangering animal health comprising alflatoxins, ochratoxins, trichothecenes, fumonisins, and zearalenone.^10^, ^12^, ^13^ As the site for toxin biodegradation, the liver appears especially susceptible to xenobiotics, and several mycotoxins are known to be associated with hepatopathy.^11^

Forages comprise the bulk of a typical diet for horses and, anecdotally, outbreaks of hepatopathy are sometimes resolved after a change of forage. Several studies have shown mycotoxins to be common in hay in Europe and the United States (USA),^14^, ^15^, ^16^, ^17^ although associations with disease have not been investigated.

We aimed to look for evidence of mycotoxicosis as a possible cause of hepatic disease in horses by comparing the prevalence of different mycotoxins in hay samples from premises known to have an outbreak of hepatopathy in resident horses versus yards with no such evidence.

## 2 METHODS

Our case control clinical study identified premises of horses within a single equine veterinary practice where client‐owned resident horses were known to have (“case premises”) or not have (“control premises”) suffered an outbreak of liver disease between December 2012 and December 2017. As soon as horses were identified for inclusion in the study, hay samples from case and control premises were collected and submitted for mycotoxin analysis using a liquid chromatography/mass spectrometry method, targeting up to 54 individual mycotoxins (Alltech 37+ Analytical Services Laboratory, Nicholasville, Kentucky [ISO/IEC 17025:2005 No. 79481, Certificate No. L14‐281 and ISO/IEC 17025:2017 No. 79481, Certificate No. L20‐392, Perry Johnson Laboratory Accreditation, Inc]) following methods described previously.^18^ Sampling was performed by selecting a handful of hay from the center of 6 to 10 stored bales randomly selected from different areas of the hay store and pooling all of the collected samples into a single plastic bag for analysis by the testing laboratory. The sample was homogenized at the testing laboratory before analysis.

A case premises was defined by at least 4 resident horses being confirmed to have liver disease contemporaneously on the basis of serum biochemistry or liver biopsy with no etiology identified The actual serum biochemical analytes measured varied among cases, but at least included gamma glutamyltransferase (GGT). Typically, an index case was identified after a veterinary examination, and serum biochemical testing requested by the horse's owner because of a clinical concern. In such index cases, testing included GGT, aspartate aminotransferase (AST) and glutamate dehydrogenase (GLDH) activities, often along with several other hepatic and nonhepatic biomarkers such as hematology, serum protein concentration, acute phase protein concentrations, alkaline phosphatase activity, bile acid concentrations, and serum creatinine and urea concentrations. After diagnosis of the index case by the attending veterinary surgeon, owners of horses also present on the same premises were advised to have serum biochemical investigations performed on their horses to determine whether or not they were part of a wider subclinical hepatopathy outbreak, as is standard procedure for this veterinary practice. Such secondary screening included at least measurement of serum GGT activity and, in some cases, also GLDH and AST activity. Case premises were included where such testing identified serum biochemical evidence of liver disease in at least 4 resident horses and at least 70% of all tested horses were determined to be affected by liver disease (ie, affected/total: 4/4, ≥4/5, ≥5/6, ≥5/7, ≥6/8, ≥14/20, etc). Control premises also were sought where there was reasonable certainty that resident horses were not affected by an outbreak of liver disease. Control premises were recruited from a large group of submissions from horses within the same veterinary practice as part of routine wellness examinations and found to have no clinical or serum biochemical evidence of liver disease. Specifically, a premise only was included as a control when at least 4 horses on the same premises had been subject to clinical examination and serum biochemical testing including serum albumin and total protein concentrations, GGT, AST, GLDH, and alkaline phosphatase activity and serum creatinine concentration, and all found to have no clinical evidence of liver disease, with at least GGT, AST, and GLDH activities within reference intervals in all tested horses. This minimum number of horses was chosen based on the observation that outbreaks of hepatopathy typically involve at least 70% of resident horses and therefore the probability of selecting a minimum of 4 unaffected horses on any affected premises would be <1% (0.3^4^).

### 2.1 Data analysis

GraphPad Prism version 9.3.1 (GraphPad Software LLC) was used for data analysis. For both case and control samples, both the prevalence and concentrations of individual mycotoxins were recorded. The frequency distribution of samples positive or negative for mycotoxins was compared between case and control premises using Fisher's exact test. Mycotoxin concentrations were compared between case and control premises using the Mann Whitney test. Significance was assumed when *P* < .05.

## 3 RESULTS

In total, 29 case premises and 12 control premises were identified and hay samples tested. In case premises, 23/29 (79%) samples were positive for mycotoxins compared to 10/12 (83%) samples from control premises (*P* > .99; relative risk [RR], 0.93; 95% confidence interval [CI], 0.64‐1.75). Median (interquartile range [IQR]) total mycotoxin concentration did not differ significantly between case and control samples (85.8 μg/kg [1.6‐268] vs. 315 μg/kg [6.3‐860]; *P* = .16).

For all 41 samples, 25 different mycotoxins were found, 10 only on case premises, 5 only on control premises and 10 on both case and control premises (Table 1).

**TABLE 1:** Mycotoxins detected in case and control samples

Mycotoxin	Positive samples			Max. concentration (μg/kg)		*P*‐value
	Cases (n = 29)	Controls (n = 12)	Total (n = 41)	Cases	Controls	
Fusaric acid	4	6	10	5.0	252.0	.004
Ochratoxin A	4	5	9	2.9	2.5	.09
Fumonisin B1	9		9	3175.0		.04
Neosolaniol	*4*	*2*	*6*	*293.0*	*687.0*	
T2		*1*	*1*		*14.1*	
HT2		*1*	*1*		*47.1*	
**Total type A trichothecenes**	4	4	8	293.0	687.0	.16
Penicillic acid	3	5	8	432.0	814.0	.02
aflatoxin B2	*2*	*1*	*3*	*10.1*	*10.9*	
aflatoxin G2	*1*	*1*	*2*	*1.7*	*232.0*	
aflatoxin B1	*1*		*1*	*3.2*		
aflatoxin G1	*1*		*1*	*55.6*		
**Total aflatoxins**	5	2	7	55.6	232.0	.78
Ergometrine	3	2	5	25.9	6.0	
15‐Acetyldeoxynivalenol	*2*		*2*	*634.2*		
deoxynivalenol	*2*		*2*	*25.5*		
nivalenol	*1*		*1*	*199.3*		
**Total type B trichothecenes**	5		5	634.2		.32
2‐bromo‐alpha‐ergocryptine	3	1	4	81.6	130.7	
Sterigmatocystin	1	1	2	1.3	2.8	
Mycophenolic acid		2	2		66.0	
Roquefortine C	1	1	2	3.0	125.8	
Zearalanone	2		2	179.5		
Fumonisin B2		1	1		348.0	
Methylergonovine	1		1	45.1		
Verruculogen	1		1	4.1		
Wortmannin	1		1	42.9		
Lysergol		1	1		152.4	

*Note*: Statistical comparison was only applied to those with >1 positive sample in each category. All median values were zero except for fusaric acid in controls (4.75 μg/mL).

Of the 29 case samples, 16 contained at least 1 of the 10 individual mycotoxins that were not found on any control premises. These comprised fumonisin B1 (9 cases), 15‐acetyldeoxynivalenol, deoxynivalenol, and zearalenone (2 cases each), aflatoxins B1 and G1, methylergonovine, nivalenol, verruculogen and wortmannin (1 case each). The median (IQR) concentration of fumonisin B1 was significantly higher in case versus control samples (0 μg/kg [0‐81.7] vs. 0 μg/kg [0‐0]; *P* = .04).

## 4 DISCUSSION

We found that mycotoxins were present in >80% of hay samples fed to horses in the United Kingdom, although neither the overall prevalence of mycotoxins nor total mycotoxin concentration differed between hay fed to horses with liver disease versus those from control premises. However, 10 different mycotoxins were found exclusively on premises with liver disease and might therefore be considered as potential causative agents. Of these, fumonisin B1 was the most prevalent and differed significantly between case and control samples, but others exclusive to case samples comprised 15‐acetyldeoxynivalenol, deoxynivalenol, zearalenone, aflatoxins B1 and G1, methylergonovine, nivalenol, verruculogen, and wortmannin.

No previous studies examined associations between forage mycotoxins and liver disease in horses. Although most studies on mycotoxin contamination have focused on cereal products, several studies have shown mycotoxins to be common in fresh grass and forage in Europe and the United States. Various Fusarium species of fungi appear to be common in pasture,^16^ and the majority of mycotoxins found in grass and hay are known to be produced mainly or exclusively by Fusarium species, including zearalenone, trichothecenes, and fumonisins.^10^ Zearalenone and the type A and type B trichothecenes, T2 and deoxynivalenol, were found to be very common in grass in the Czech Republic, especially in July, with lower concentrations in June and December.^19^, ^20^ The mass of these mycotoxins was found to increase further during storage of the grass as silage.^19^ Similarly, examination of rye grasses in Germany found zearalenone in 67% of samples, and the type A trichothecenes T2 and diacetoxyscirpenol in 25% and 22% of samples, respectively.^16^ A more recent study from Germany also found zearalenone to be the most common mycotoxin in hay (43% of samples), with the type B trichothecenes nivalenol, deoxynivalenol and 3‐acetyldeoxynivalenol also found in lesser quantities.^15^ In Ireland, examination of 149 hay samples, grown locally and imported from Canada, found only zearalenone as a mycotoxin contaminant.^14^ It was found in 21% of Irish hay samples but in only 8% of Irish haylage or imported Canadian hay. Thus, zearalenone appears to be the most common mycotoxin in European hays, with trichothecenes also often present. In contrast, a study of hays and silages in Minnesota, Wisconsin, and Illinois in the United States failed to detect any zearalenone although *Alternaria alternata* TA toxin, cyclopiazonic acid, deoxynivalenol, fumonisin B1 and roquefortine all were very commonly found.^17^

The most common mycotoxins found in hay in our study were fusaric acid, ochratoxin A, fumonisin B1, penicillic acid, and neosolaniol (Table 1). The only mycotoxin found in forage from case premises in significantly higher concentrations than in control samples was fumonisin B1. In contrast, fusaric and penicillic acids were found in significantly larger amounts in control hay samples, with ochratoxin A almost reaching significance (Table 1). This finding may reflect influential storage and growth factors favoring production of specific mycotoxins with and without hepatotoxic potential. Mycotoxin contamination is known to vary significantly for the same crop in different years, depending on factors such as local temperature and humidity.^19^ Interestingly, despite fusaric acid, penicillic acid or ochratoxin A being present in 20/41 (49%) hay samples, only 1 of the 9 samples positive for fumonisin B1 contained fusaric acid (the lowest detected amount of all samples) and none contained penicillic acid or ochratoxin A, perhaps suggesting important differences in storage parameters for hay on case and control premises.

Adverse health consequences of mycotoxins can be complex and unpredictable and depend on mycotoxin concentration, chronicity, animal species, bioavailability and co‐exposure to other mycotoxins.^10^, ^21^, ^22^ In addition to the association of fumonisin B1 with liver disease in our study, it is possible that other mycotoxins also might have pathogenetic relevance. Evidence of interactions among several hepatotoxic mycotoxins is reported including aflatoxin B1, deoxynivalenol, nivalenol, T2, fumonisin B1, zearalenone, and moniliformin.^23^, ^24^ Interestingly, although less commonly found than fumonisin B1 in our study, aflatoxin B1, 15‐acetyldeoxynivalenol, deoxynivalenol, nivalenol, and zearalenone also were found exclusively in hay samples from case premises (Table 1).

Fumonisin B1 acts as a competitive inhibitor of ceramide synthase and thus inhibits the sphingolipid biosynthetic pathway.^25^ Horses are considered to be more sensitive to fumonisin than other species and neuro‐ and hepato‐toxicity are the main consequences.^26^, ^27^ Fumonisin B1 is best known in equine medicine as the cause of leukoencephalomalacia in association with ingestion of moldy corn.^28^ However, an outbreak of equine leukoencephalomalacia in horses also has been reported after consumption of fumonisin‐contaminated forage.^29^ Fumonisin B1 also is known to cause hepatotoxicity in many species including rats,^30^ mice,^31^ rabbits,^32^ pigs,^33^ calves,^34^ chickens,^35^ and horses.^27^, ^28^, ^29^, ^36^ Although acute hepatotoxicity with centrolobular necrosis, cytoplasmic vacuolation, biliary hyperplasia and moderate to severe portal fibrosis is recognized in association with ingestion of large amounts of fumonisin B1 in horses,^27^, ^28^, ^29^, ^36^ the possibility and nature of toxicity resulting from chronic ingestion of smaller amounts has not been well investigated. Studies of fumonisin B1 toxicity in rodents generally have found hepatocyte apoptosis as a prominent feature along with some necrotic hepatocytes.^37^ Interestingly, although not a specific finding, scattered individual hepatocyte necrosis and apoptosis are the most common histopathologic features in liver biopsy specimens collected from horses involved in outbreaks of liver disease in the United Kingdom (A.E. Durham, unpublished observations).

Most cases of leukoencephalomalacia in horses have been associated with feeds containing >10 ppm fumonisin B1, and it is recommended that feeds for horses contain no more than 1 to 5 ppm fumonisin B1.^27^ In this respect, only 1 of the cases in our study had a concerning value (3.175 mg/kg), although it cannot be assumed that any toxin was evenly distributed in the forage and it remains possible that higher concentrations may have been present in other areas of the stored product, or even in hay that had already been consumed. It is possibly more relevant that identifying the presence of certain toxins in the hay indicates the presence of relevant toxinogenic fungi and also appropriate environmental conditions for toxinogenesis. Thus, the qualitative presence of toxins, rather than their quantified amount in hay, might have more relevance in similar outbreaks.

Various approaches have been used to attempt to decrease mycotoxin presence in feeds using processing or additives,^9^, ^38^ although only some would be feasible for forage treatment. Heat may destroy mycotoxins such as fumonisin B1, alflatoxin B1, deoxynivalenol, nivalenol, and zearalenone although often temperatures >150°C are required for a consistent effect, which is probably beyond the range of most hay steamers.^39^, ^40^, ^41^ Some mycotoxins such as fumonisin B1, deoxynivalenol, and nivalenol are highly water soluble and may be removed effectively by washing or soaking whereas others such as alflatoxins, zearalenone, and type A trichothecenes are more hydrophobic.^42^ Many potential adsorbents are available with variable efficacy for removal of mycotoxins. Silicates such as kaolin, bentonite, montmorillonite and zeolite are the most widely used although others exist, and charcoal, yeast (including Saccharomyces cerevisiae), and bacteria (including Lactobacilli) also have been studied.^9^ Another approach to mitigating the effect of mycotoxins includes nutritional supplements to modulate detoxification of mycotoxins or to counteract their toxic effects. One study found that glucose reactants of fumonisin were far less toxic in pigs.^33^ Various vitamins and other micronutrients such as vitamin E, selenium, methionine, glutamic acid, arginine, aspartate, and lysine have been investigated in this respect.^9^ Different grass species appear to have different susceptibilities to fungal colonization and mycotoxin production, offering a means of control when mycotoxicosis occurs.^19^, ^20^, ^43^, ^44^ Fertilization of pasture also might decrease mycotoxin contamination in some cases.^44^

The major limitation of our study was the potential for sampling error. Cut herbage from different parts of the same field often vary in plant content, moisture and fungal contamination. Furthermore, storage of hay within different parts of the same bale or larger scale stack could introduce further marked variability in preservation, moisture and temperature of the forage. Thus, submitted forage samples may not have been truly representative of the forage consumed on the premises. Indeed, it might even have been that any hepatotoxic forage had already been consumed, leaving qualitatively different forage for analysis. Nevertheless, the presence of specific mycotoxins found in our study at least establishes that the criteria for toxinogenesis were fulfilled in specific samples. The study was further limited by availability of premises for inclusion, especially control premises. Thus, statistical power was suboptimal and the descriptive findings simply provide a basis for further study of certain mycotoxins. In particular, mycotoxins that were found more commonly in control premises (eg, fusaric acid, ochratoxin, penicillic acid) would appear unlikely to have relevance to hepatopathy outbreaks and focus on other mycotoxins such as fumonisin B1 would appear more logical (Table 1).

Our study was based on the hypothesis that forage‐associated mycotoxins might be a cause of liver disease outbreaks in horses in the United Kingdom. Although the study did not establish causal associations between mycotoxins and hepatopathy, interesting associations between liver disease and mycotoxins with known hepatotoxic potential were found, providing a basis for further investigation of any putative links.

## CONFLICT OF INTEREST DECLARATION

Author declares no conflict of interest.

## OFF‐LABEL ANTIMICROBIAL DECLARATION

Author declares no off‐label use of antimicrobials.

## INSTITUTIONAL ANIMAL CARE AND USE COMMITTEE (IACUC) OR OTHER APPROVAL DECLARATION

Author declares no IACUC or other approval was needed.

## HUMAN ETHICS APPROVAL DECLARATION

Author declares human ethics approval was not needed for this study.
